# Impact of Social Determinants of Health in the Treatment of Closed Nasal Bone Fractures

**DOI:** 10.3390/cmtr19010004

**Published:** 2026-01-08

**Authors:** Nicholas A. Frisco, Nicholas W. Clark, Kayla W. Kilpatrick, Maragatha Kuchibhatla, David B. Powers, Charles R. Woodard, Nosayaba Osazuwa-Peters, Dane M. Barrett

**Affiliations:** 1 Department of Head and Neck Surgery & Communication Sciences, Duke University, Durham, NC 27710, USA; naf23@duke.edu (N.A.F.); david.powers@duke.edu (D.B.P.); charles.woodard@duke.edu (C.R.W.); nosa.peters@duke.edu (N.O.-P.); 2 Premier Image Cosmetic & Laser Surgery, Atlanta, GA 30328, USA; nwclark22@gmail.com; 3 Department of Biostatistics & Bioinformatics, Duke University, Durham, NC 27710, USA; kayla.kilpatrick@duke.edu (K.W.K.); maragatha.kuchibhatla@duke.edu (M.K.); 4 Division of Plastic, Maxillofacial, and Oral Surgery, Duke Department of Surgery, Durham, NC 27710, USA; 5 Department of Population Health Sciences, Duke University School of Medicine, Durham, NC 27710, USA; 6 Duke Cancer Institute, Duke University, Durham, NC 27710, USA

**Keywords:** craniomaxillofacial trauma, nasal fracture, social determinants of health

## Abstract

Study Design: Retrospective cohort study. Objective: To determine the association of social determinants of health with rates of closed nasal bone reduction. Methods: A retrospective analysis of the National Trauma Data Bank (NTDB) from 2011 to 2019 was performed, including only adult patients with isolated nasal bone fractures. Logistic regression modeling was used to estimate the association between closed nasal bone reduction and sociodemographic variables. Results: A total of 149,312 patients were included, with an average age of 50. Most patients were male (68%), White (72%), and non-Hispanic/Latino (77%), with Medicare insurance (25%). Most patients were cared for at non-university (54%) and non-profit hospitals (88%). A total of 39% were cared for at an ACS level 1 trauma center. Finally, 3.3% of the patients in this study underwent closed reduction. The odds of undergoing reduction decreased with increasing age (OR: 0.99, CI: (0.99, 0.99)). Compared to White patients, Asian and Black/African American patients had decreased odds of closed reduction (Asian: OR (CI) 0.71 (0.53, 0.95); Black: OR (CI): 0.71 (0.65, 0.79)). Patients with government insurance or who were uninsured had lower odds of closed reduction compared to private/commercial insurance, with Medicaid, Medicare, and not billed/self-pay odds ratios of 0.83 (CI: (0.76, 0.90)), 0.81 (CI: (0.73, 0.89)), and 0.79 (CI: (0.72, 0.86)), respectively. Conclusions: Social determinants of health are associated with differential rates of inpatient closed nasal bone reduction. Further studies in the outpatient setting are needed to determine if these associations remain consistent.

## 1. Introduction

There are an estimated 28,394 new cases of nasal bone fractures annually in the United States, making nasal bone fractures the most common facial fracture [1]. The cost of management of nasal bone fractures in the United States healthcare system has risen by 78% in the last decade [1], even though most nasal bone fractures may not require intervention [2]. The decision to intervene is a complex one with many factors to consider, including the severity of the fracture and its effects on the function and aesthetics of the nose [3]. Despite the high incidence of nasal fractures after facial injury, as little as 7.5% of nasal bone fractures undergo closed reduction [4]. Several clinical factors have been shown to contribute to the low overall rate of closed reduction for nasal bone, including lack of cosmetic or functional airway impact of minimally displaced injuries, severe concomitant facial injury requiring open approach, and medical comorbidity precluding the ability to undergo a timely procedure [5,6]. In one study, increased overall injury severity was associated with up to 30% greater odds of undergoing reduction, while older age and medical comorbidity, such as myocardial infarction or dementia, was associated with significantly decreased odds of reduction [7]. Significant delay in care after injury or lack of follow-up care may also influence the overall rate of reduction in nasal fractures, as reduction is ideal within a two-week window following injury before bone healing occurs [5]. As such, non-clinical factors appear to play a significant role in management patterns of nasal fractures. While increased socioeconomic deprivation and sex have been shown to correlate with interpersonal violence and severe facial injury in prior studies globally [8,9,10], contextual factors such as lack of comprehensive insurance coverage and rural residence have also been associated with delays in care of facial injury [11,12]. Access to institutions equipped with specialized craniomaxillofacial (CMF) care has also been shown to be a bottleneck in the care paradigm, particularly in resource-limited countries [12]. This may also limit the availability of closed reduction in the emergency department at index presentation. Though most nasal bone reductions occur in the outpatient setting [13], there is a large proportion of patients with limited access to CMF follow-up care in general that may not be able to adhere to an outpatient management protocol [14,15].

The United States Department of Health and Human Services, Office of Disease Prevention and Health Promotion (ODPHP), recognizes that social determinants of health (SDOH), upstream factors outside of health care delivery, influence patient care and contribute to health disparities [16]. It is vital to understand the nature and influence of these SDOH so that subjectivity in provider decision-making and attrition in follow-up care can be minimized. Disparities in healthcare delivery and outcomes have been well-defined with respect to trauma patients [17], and in the general and orthopedic trauma literature, race, age, education, housing, occupation, and distance from hospital have been associated with lower utilization of outpatient resources [17,18]. Unfortunately, the current literature examining the potential impact of SDOH on nasal bone fracture care and outcomes is limited. To address this gap in the literature, we aimed to explore whether SDOH increased or decreased the odds of receiving a closed nasal bone reduction.

## 2. Methods

### 2.1. Data Source and Study Sample

We used the National Trauma Data Bank (NTDB), a de-identified database containing over 7.5 million records of trauma data from more than 900 US trauma centers, managed by the American College of Surgeons (ACS) [19,20]. The NTDB was used to perform a retrospective analysis on data from 2011 to 2019 on patients with isolated closed nasal bone fractures. This database used de-identified patient data and was exempt from the Institutional Review Board at Duke University. International Classification of Diseases, 9th revision (ICD-9) or 10th revision (ICD-10) codes, were used to identify patients with closed nasal bone fractures (ICD-9 code: 802.0 for data from 1 October 2015 or older and ICD-10 code: S02.2XXA for data from 2 October 2015 and later).

### 2.2. Measures

The outcome of interest was the receipt of closed nasal bone reduction in patients with isolated closed nasal bone fractures. Male and female patients 18 years of age or older with closed nasal bone fractures were included in this study. Notably, injury ICD-9 and ICD-10 codes were assigned preferentially based on operative reports, radiology reports, physician notes, trauma flowsheets, history and physical notes, nursing flowsheets, progress notes, and discharge summaries, respectively [19]. Patients with any facial fracture other than nasal bone fracture (excluded via ICD codes), open nasal bone fracture, and patients dead on arrival to the emergency department were excluded. Patient characteristics, including age, gender, race, insurance status, and injury severity score (ISS), were collected. Abbreviated Injury Score (AIS) was calculated based on a six-point scale for each anatomical location, which is compiled to make up the ISS [21]. There is no validated measure of nasal injury severity available in NTDB; therefore, a variable denoted as “nasal summary AIS count” was created. Nasal summary AIS count uses the number of nasal AIS codes per person to serve as a proxy for nasal injury severity. A larger count is thought to represent a more severe injury. Additional variables, including hospital teaching status, hospital type, and trauma center status (ACS designation), were collected and analyzed. ICD-9 and ICD-10 procedure codes were used to identify patients who received closed nasal fracture reduction. According to NTDB, these codes captured procedures performed in the operating room, emergency department, ICU, or ward during the patient’s index hospital encounter [19].

### 2.3. Statistical Analyses

Patient demographics and other characteristics were presented as descriptive statistics, with chi-square or Kruskal–Wallis tests used for comparisons of the characteristics between patients who underwent closed reduction and those who did not. The primary analysis was conducted using logistic regression, modeling the probability that closed reduction was performed. Goodness-of-fit was assessed, and the C-index was reported for the fitted model. This model excluded individuals missing gender information or hospital type, as there were cell counts of zero in the closed reduction group for both variables, leading to poor estimation of these variables. All other variables with missing data included “missing” as a separate category, with this category having a sufficient cell count for estimation according to full information maximum likelihood under the assumption that the data in NTDB is missing at random. In this way, bias associated with complete-case deletion was avoided and statistical power preserved where possible. The estimates for these categories are not interpreted. All statistical analyses were performed using SAS 9.4 (SAS Institute Inc., Cary, NC, USA).

## 3. Results

A total of 149,312 patients in the NTDB from the respective years were included in the study, with an average age of 50. Most patients were male (68%), White (72%), and non-Hispanic/Latino (77%), with Medicare insurance (25%). The mean ISS was 10.3 with a standard deviation of 8.8. Most patients had 1 nose AIS code (93%), were cared for at a non-university hospital (54%) and non-profit hospitals (88%), and were at an ACS level 1 trauma center (39%).

Table 1 displays the demographics of patients in this dataset stratified by whether a closed nasal bone reduction was performed. In total, 3.3% of the patients in this study underwent closed reduction. Patients who underwent reduction tended to be younger than those who did not, with a mean age of 45 (SD: 19) compared to a mean age of 50 years (SD: 21).

ISS was similar for those who did and those who did not have closed reductions. There was a mean of 10.5 (SD 8.3) for those with closed reductions and 10.3 (SD 8.8) for those who did not undergo closed reduction. There were higher percentages of patients who had closed reduction compared to those who did not when the nasal summary AIS count is greater than or equal to two, indicating a more severe injury.

Table 2 provides the results of the multivariable logistic regression model predicting closed reduction in nasal bone fractures. Age was associated with closed reduction, with the odds of having a reduction decreasing as age increases (OR: 0.99, CI: (0.99, 0.99)). Females had 1.09 times the odds of having a closed reduction compared to males (CI: (1.02, 1.16)). Overall, race was associated with closed reduction in nasal bone fracture (type 3 Wald test), and compared to White patients, Asian and Black/African American patients had decreased odds of closed reduction (Asian: OR (CI) 0.71 (0.53, 0.95); Black: OR (CI): 0.71 (0.65, 0.79)). Hispanic/Latino patients had increased odds of closed reduction compared to those who were not Hispanic or Latino. However, Hispanic/Latino ethnicity was not predictive of closed reduction compared to non-Hispanic/Latino with an odds ratio of 1.10 (CI: (1.00, 1.21)). Overall, insurance was associated with closed reduction in nasal bone fracture (type 3 Wald test). Medicaid, Medicare, and not billed/self-pay had decreased odds and were predictive of closed reduction compared to private/commercial insurance. At the same time, other government insurance had increased odds but were not predictive of closed reduction compared to private/commercial insurance. Medicaid, Medicare, and not billed/self-pay had odds ratios of 0.83 (CI: (0.76, 0.90)), 0.81 (CI: (0.73, 0.89)), and 0.79 (CI: (0.72, 0.86)), respectively. Hospital teaching status was associated with closed reduction; non-university hospitals had increased odds of closed reduction compared to university hospitals, with an odds ratio of 1.21 (CI: (1.13, 1.29)). Hospital type was predictive of closed reduction, with for-profit hospitals having increased odds of closed reduction compared to government/non-profit hospitals (OR (CI): 1.19 (1.09, 1.30)). The ACS designation was associated overall with closed reduction. Both designations II and III/IV had decreased odds of closed reduction compared to designation I (II: OR (CI): 0.75 (0.69, 0.81); III/IV: OR (CI): 0.32 (0.25, 0.40)). Overall, the nasal summary AIS count was associated with closed reduction (type 3 Wald test). Having 2 or 3+ nose AIS codes, indicating more nasal injuries, was predictive of closed reduction, with these categories having increased odds of closed reduction compared to having 1 nose AIS code (2 codes: OR (CI) 2.39 (2.19, 2.62); 3+ codes: OR (CI) 2.80 (1.65, 4.76).

**Figure 1:**
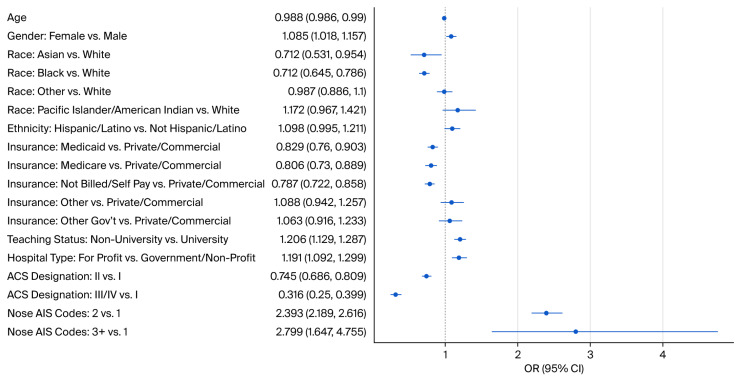
Odds ratios (OR) and confidence intervals (CI) for associations between social determinants of health and closed nasal bone reduction.

Figure 1 displays odds ratios and confidence intervals between social determinants of health and closed nasal bone reduction. An odds ratio greater than 1 indicates increased odds of having closed reduction, while an odds ratio less than 1 indicates decreased odds of having closed reduction. For categorical factors, odds ratios are compared to the reference group.

## 4. Discussion

This study aimed to build upon data reported by Pham and colleagues [4] and further evaluate the impact of social determinants of health on facial trauma care, specifically on closed nasal bone reduction. We demonstrated that SDOH such as age, gender, race and insurance status were significant factors in predicting whether a patient underwent closed reduction during the index hospital encounter. We also found additional factors, such as hospital type, teaching status, ACS trauma designation, and nasal summary AIS count, were significant factors in predicting whether a patient underwent closed reduction in these injuries.

Our study found that 3.3% of the 149,312 patients with isolated nasal bone fractures underwent reduction. This reduction rate is lower than reported by Pham and colleagues [4], who reported 5.2% of 131,967 patients. This is partly explained by different NTDB years evaluated (2007–2015 vs. 2011–2019). However, age (50 vs. 47), percentage male (68% vs. 71%), along with other characteristics, were much closer in our study to aforementioned study [4]. This may indicate that rates of closed nasal bone reduction is declining at least in the immediate setting. This could be explained by an increasingly greater number of nasal fracture reductions occurring in the outpatient setting, which are not captured in NTDB [13]. As facial fractures are increasingly recognized as injuries that can be managed safely in the follow-up setting [22], further studies are needed to assess closed nasal bone reduction in the outpatient setting. As demonstrated by Stewart and colleagues [14], non-clinical factors such as race and lack of basic resources are significantly associated with poor follow-up adherence, highlighting that SDOH and access to care considerations may significantly influence disparate utilization of outpatient resources. However, with the majority of isolated facial fractures in the United States being repaired in the outpatient setting [23], it is important to consider how SDOH may have played a role in the upfront decision to repair isolated nasal bone fractures in an inpatient setting. In contrast to management decisions driven by injury severity, such as increased reduction rates with increased nasal AIS, as we demonstrated, the effect of patient contextual factors has been less apparent in prior studies. In a study by Wasicek and colleagues [23] utilizing NTDB from 2007 to 2015, age, sex, insurance status, race and hospital type were all independently associated with differing rates of early operative intervention. However, individual fracture patterns were not examined, and patients with multiple facial fractures were included. Unlike injury to the mandible and lower third, where treatment paradigms have been more closely associated with anatomic subunit than SDOH [24], nasal bone fracture management is more influenced by subjective factors such as aesthetic considerations, patient-specific factors and preferences, as some studies have shown that preoperative functional nasal obstruction rates can be as low as 20.4% despite accompanying deformity [25]. Prior studies utilizing the American College of Surgeons Trauma Quality Improvement Program (ACS-TQIP) database have determined that race, sex, insurance, and setting were associated with reduced odds of specifically nasal fracture reduction and delays in intervention in the inpatient setting [7,11], but these studies also included patients with multiple facial fractures, often contributing to prolonged hospitalizations. This is the first known study to evaluate the impact of SDOH on the treatment of isolated nasal bone injuries during the index hospital encounter.

Compared to White individuals, Asian and Black/African American individuals were less likely to undergo inpatient closed nasal bone reduction in our study. These findings agree with the aforementioned ACS-TQIP study [7], who found that Black patients were less likely to undergo inpatient nasal fracture reduction. With exclusion of multiple facial injuries, our study may have controlled for severe mechanisms of injury, which have been disproportionate presentations according to race and SES in the literature [26,27]. For example, African American patients have been shown to more frequently experience traumatic mechanisms with a higher risk of death [28], likely injuries that would not be electively repaired, but this association is less likely to contribute to our findings compared to prior study [7]. This highlights important contextual factors, apart of injury and exposure, that must be considered when examining the association of race with receipt of trauma care. Considerations such as access to care, distance from hospital, limited ability to miss work, trust in the healthcare system, and provider implicit bias may play a role in the disproportionate decisions to discharge without closed reduction. Comorbidity burden has also been associated with race and practice setting, which can contribute to the decision to offer upfront closed reduction [29]. On a broader scope, our findings are consistent with trends in surgical care utilization in the United States [30]. However, only inferences can be made, and future study is needed to explore these health differences in more granular detail.

Our study found that patients with Medicare, Medicaid, and uninsured patients were less likely to undergo closed reduction in nasal fractures compared to patients with private/commercial coverage during the index hospital encounter. Although our findings regarding uninsured patients agree with prior analysis of national data, our finding that patients with public insurance were less likely to undergo reduction in nasal fractures during the index hospital encounter contrast with prior study in the same setting [7]. Paliwoda and colleagues [7] found that public insurance holders were more likely to undergo reduction and postulated that these patients may experience broader care coverage, fewer out-of-pocket costs and more incentivized providers, given reimbursement patterns. The contrasting findings might be explained by the exclusion of more severely injured patients with longer hospital stays, which have been more prevalent among public insurance carriers in the broader trauma literature [31,32]. In general, public insurance has been associated with lower likelihood of treatment in high-quality, specialized centers, and decreased receipt of procedures [32,33]. As with other non-clinical variables, insurance status may also be confounded by other factors in a patient’s context that are incompletely captured in national datasets, leading to incongruent conclusions on reported health differences in the literature. However, these data support the findings of prior studies related to insurance and the receipt of trauma care overall [32].

Patients had increased odds of closed reduction at ACS level I trauma centers in the current study, which could be attributable to dedicated on-call facial reconstruction teams with more flexible OR availability [34]. These findings agree with prior analyses of national analyses of facial injuries, and, further, this point is of critical importance in low-resource countries, where intervention may not be feasible locally due to lack of CMF-trained staff outside of specialized tertiary referral centers [12]. However, the finding that nasal bone reductions during the index hospital encounter have increased odds of occurring in for-profit hospitals is not readily explained, especially given that outpatient elective management of isolated facial fractures has been demonstrated to be a cost-effective management paradigm [35]. One consideration is that safety net hospitals often provide greater access to early intervention, and some of these institutions are for-profit, though this likely represents a minority of cases.

Regarding injury severity, Pham and colleagues [4] previously reported ISS of those with isolated nasal bone fracture but did not evaluate whether this predicted closed reduction. In order to capture the severity of injury to the nose most accurately, we used the AIS specific to the nose and demonstrated increased number of nasal specific injuries predicted increased odds of closed reduction. The nasal summary AIS count per patient was used to serve as a proxy for nasal injury severity since each individual nose AIS alone was rated a low overall injury severity. The ISS is the sum of squares of the highest AISss in the three most injured bodily segments, but given its utilization of the highest score per subsite, a drawback of the metric is its limited ability to account for multiple injuries in one body region [21,36,37]. Conventionally, ISS has not been able to account for the implications of multiple injuries in the same body region, particularly in head-injured patients [37]. With the current iteration of the AIS, more than one code is often assigned to a given body region, for example in sites with external soft tissue and internal bone or visceral injury [37]. In the context of isolated nasal injuries examined in our study, a higher number of assigned codes was thought to represent a more severe injury. This variable was used in the primary model for closed nasal bone reduction because the severity of the nasal injury should influence procedural intervention. We excluded any patients with facial or head injuries but did not exclude those patients with injuries in other parts of the body. Gennarelli and colleagues [21] demonstrated when AIS score is lower, outcomes are determined by factors other than survival or mortality and further demonstrated that at AIS of 1 and 2 (meaning low severity), mortality is so low that little mortality/survival differences exist, meaning low severity injuries will likely not lead to mortality. ISS has been shown to correlate strongly with mortality in trauma patients [38] but in isolated nasal bone fractures this score is less helpful [39]. Furthermore, particularly for low mortality injuries, it has been postulated that further granularity in coding may improve discriminatory ability of the measure [36]. To account for the current limitations in the ISS and AISs, we used the nasal summary AIS count as our measure of injury severity. Understanding its limitations with respect to interobserver variability of assigned codes and lack of standard validation, the conclusions regarding injury severity in our study may not be generalizable and complexity of associated injuries may be misclassified.

This study has several other limitations. One of the most significant limitations of this study exists in the use of the data sample captured by the NTDB. Closed nasal fractures were identified according to a data hierarchy based on operative reports, radiology notes, and physician and nursing notes, as defined in the methods. In the data, there is no distinction of whether imaging was used to confirm the diagnosis or if the fractures represented chronic untreated injury. Therefore, potential misclassification bias exists whereby the true number of acute nasal injuries that would be candidates for reduction could be overestimated. Patients treated on an outpatient basis, who represent a large proportion of those with nasal bone fractures, are not captured in the NTDB. As the procedure codes captured by NTDB represent interventions performed in the operating room, ward, ICU, and emergency department, patients treated in the clinic during a follow-up encounter were not included in the data set, and this may underestimate the influence of SDOH on the treatment of nasal bone fractures, particularly given the previously discussed effects of SDOH on follow-up care. Reporting bias also exists in the current study, as not all hospitals caring for nasal bone fractures report to the NTDB. However, a sizeable number of patients each year still receive inpatient closed reduction for isolated nasal bone fractures. Examining differences in healthcare utilization among this population is valuable, especially considering the implications on repair timing related to inpatient status for CMF patients overall [40]. Our findings are interesting; however, as healthcare differences according to sociodemographic factors would be expected to be more evident in the outpatient setting, where access to care issues associated with that location amplify these differences. Our findings suggest that non-clinical factors may also play a significant role in inpatient management patterns.

Furthermore, data regarding the involvement of specialty consultants is not known (otolaryngology, plastic surgery, and oral and maxillofacial surgery). The distribution of specialty services at level I trauma centers was evaluated previously by Bagheri and colleagues [41], and Cohn and colleagues [42] showed there were statistically significant differences in management offered, though not in outcomes. Ultimately, this study examines a robust national data set that is powered to assess health differences concerning both injury severity patterns and patient contextual factors as they relate to the receipt of closed nasal bone reduction for isolated nasal bone fractures during the index hospital encounter.

## 5. Conclusions

In conclusion, a sizeable population of patients suffering from isolated nasal bone fractures receives inpatient operative closed reduction in their injuries. In addition to injury severity, this study found that SDOH and sociodemographic considerations are significantly associated with the differential receipt of closed nasal bone reduction. Specifically, Black and Asian race, public or lack of insurance, and treatment in facilities apart from ACS level 1 trauma centers were factors associated with lower odds of receiving closed nasal bone reduction in this setting. Further quantitative and qualitative studies are needed to explore the underlying driving factors behind these health differences.

## Figures and Tables

**Table 1:** Demographics.

	No Closed Reduction (N = 144,349)	Closed Reduction (N = 4963)	Total (N = 149,312)
Age			
N	144,349	4963	149,312
Missing	0	0	0
Mean (SD)	50.3 (20.8)	45.2 (19.4)	50.2 (20.8)
Median	49.0	42.0	49.0
Q1, Q3	32.0, 67.0	28.0, 59.0	31.0, 67.0
Range	(18.0–89.0)	(18.0–89.0)	(18.0–89.0)
Gender			
Female	45,991 (31.9%)	1520 (30.6%)	47,511 (31.8%)
Male	98,328 (68.1%)	3443 (69.4%)	101,771 (68.2%)
Missing	30 (0.0%)	0 (0.0%)	30 (0.0%)
Race			
Asian	1841 (1.3%)	47 (0.9%)	1888 (1.3%)
Black or African American	17,725 (12.3%)	478 (9.6%)	18,203 (12.2%)
Other Race	14,140 (9.8%)	583 (11.7%)	14,723 (9.9%)
Pacific Islander or American Indian	2476 (1.7%)	118 (2.4%)	2594 (1.7%)
White	103,915 (72.0%)	3607 (72.7%)	107,522 (72.0%)
Missing	4252 (2.9%)	130 (2.6%)	4382 (2.9%)
Ethnicity			
Hispanic or Latino	18,493 (12.8%)	779 (15.7%)	19,272 (12.9%)
Not Hispanic or Latino	111,910 (77.5%)	3724 (75.0%)	115,634 (77.4%)
Missing	13,946 (9.7%)	460 (9.3%)	14,406 (9.6%)
Insurance Status			
Medicaid	22,209 (15.4%)	774 (15.6%)	22,983 (15.4%)
Medicare	36,355 (25.2%)	865 (17.4%)	37,220 (24.9%)
Not Billed/Self Pay	23,541 (16.3%)	798 (16.1%)	24,339 (16.3%)
Other	5098 (3.5%)	218 (4.4%)	5316 (3.6%)
Other Government	4685 (3.2%)	208 (4.2%)	4893 (3.3%)
Private/Commercial Insurance	46,871 (32.5%)	1920 (38.7%)	48,791 (32.7%)
Missing	5590 (3.9%)	180 (3.6%)	5770 (3.9%)
Injury Severity Score			
N	141,261	4817	146,078
Missing	3088	146	3234
Mean (SD)	10.3 (8.8)	10.5 (8.3)	10.3 (8.8)
Median	9.0	9.0	9.0
Q1, Q3	5.0, 14.0	5.0, 14.0	5.0, 14.0
Range	(1.0–75.0)	(1.0–75.0)	(1.0–75.0)
Nasal Summary AIS Count			
1 code	134,857 (93.4%)	4334 (87.3%)	139,191 (93.2%)
2 codes	7937 (5.5%)	596 (12.0%)	8533 (5.7%)
3 codes	184 (0.1%)	15 (0.3%)	199 (0.1%)
4 codes	1 (0.0%)	0 (0.0%)	1 (0.0%)
Missing	1370 (0.9%)	18 (0.4%)	1388 (0.9%)
Hospital Teaching Status			
Non-University	78,256 (54.2%)	2712 (54.6%)	80,968 (54.2%)
University	65,760 (45.6%)	2245 (45.2%)	68,005 (45.5%)
Missing	333 (0.2%)	6 (0.1%)	339 (0.2%)
Hospital Type			
For Profit	17,039 (11.8%)	644 (13.0%)	17,683 (11.8%)
Government	127 (0.1%)	2 (0.0%)	129 (0.1%)
Non-Profit	127,077 (88.0%)	4317 (87.0%)	131,394 (88.0%)
Missing	106 (0.1%)	0 (0.0%)	106 (0.1%)
ACS Designation			
I	56,276 (39.0%)	2280 (45.9%)	58,556 (39.2%)
II	32,829 (22.7%)	1110 (22.4%)	33,939 (22.7%)
III	5671 (3.9%)	77 (1.6%)	5748 (3.8%)
IV	53 (0.0%)	0 (0.0%)	53 (0.0%)
Not Applicable	33,262 (23.0%)	1032 (20.8%)	34,294 (23.0%)
Missing	16,258 (11.3%)	464 (9.3%)	16,722 (11.2%)

**Table 2:** Multivariable logistic regression OR estimates for social determinants of health. Probability modeled: closed reduction in nasal bone fracture occurred.

Predictor	OR *	95% CI
Age	0.99	(0.99, 0.99)
Gender (ref: Male)		
Female	1.09	(1.02, 1.16)
Race (ref: White)		
Asian	0.71	(0.53, 0.95)
Black/African American	0.71	(0.65, 0.789)
Other	0.99	(0.89, 1.10)
Pacific Islander/American Indian	1.17	(0.97, 1.41)
Missing	0.81	(0.67, 0.97)
Ethnicity (ref: Not Hispanic/Latino)		
Hispanic/Latino	1.10	(1.00, 1.21)
Missing	1.06	(0.96, 1.17)
Insurance (ref: Private/Commercial)		
Medicaid	0.83	(0.76, 0.90)
Medicare	0.81	(0.73, 0.89)
Not Billed/Self Pay	0.79	(0.72, 0.856)
Other	1.09	(0.94, 1.26)
Other Government	1.06	(0.92, 1.23)
Missing	0.83	(0.71, 0.97)
Hospital Teaching Status (ref: University)		
Non-University	1.21	(1.13, 1.289)
Missing	0.79	(0.35, 1.79)
Hospital Type (ref: Government/ Non-Profit)		
For Profit	1.19	(1.09, 1.30)
ACS Designation (ref: I)		
II	0.75	(0.69, 0.81)
III/IV	0.32	(0.250, 0.40)
Not Applicable/Missing	0.71	(0.66, 0.76)
Nasal Summary AIS Count (ref: 1 code)		
2 codes	2.39	(2.19, 2.62)
3+ codes	2.80	(1.65, 4.76)
Missing	0.41	(0.25, 0.65)

* Type 3 Wald Test. N = 149,176. Hosmer and Lemeshow Goodness of Fit Chi-Squared Test Statistic = 7.95, df = 8, *p*-value = 0.438. C-index = 0.621. OR = odds ratio. CI = confidence interval.

## Data Availability

Data supporting this study are available from the National Trauma Data Bank (NTDB) and are for purchase from the American College of Surgeons (ACS) via https://www.facs.org/quality-programs/trauma/tqp/center-programs/ntdb/datasets. URL accessed on 10 October 2024.
